# Investigate the improvement of facial skin texture with the VISIA system after total thyroidectomy

**DOI:** 10.1186/s12893-021-01108-3

**Published:** 2021-02-21

**Authors:** Spencer C. H. Kuo, Faye Huang, Shun-Yu Chi, Hui-Ping Lin, Peng-Chen Chien, Ching-Hua Hsieh

**Affiliations:** 1 Department of Plastic Surgery, Kaohsiung Chang Gung Memorial Hospital, No.123, Ta-Pei Road, Niao-Song District, Kaohsiung, 833 Taiwan; 2 Department of General Surgery, Kaohsiung Chang Gung Memorial Hospital, Kaohsiung, Taiwan

**Keywords:** Hyperthyroidism, Thyroidectomy, VISIA

## Abstract

**Background:**

During clinical practice we have noticed that some patients with hyperthyroidism have finer skin with less wrinkles, pores, and spots after thyroidectomy, and the improvement can be observed within a few weeks after the operation. However, there is no evidence or study in the literature to proof this finding.

**Aim and objective:**

This study was designed to evaluate and quantify the skin characters of patients with hyperthyroidism before and after thyroidectomy.

**Material and methods:**

This is a prospective study to include patients with hyperthyroidism who received total thyroidectomy between March 1st, 2018 and February 28th, 2019. The patients received blood test for T4 and TSH analysis and VISIA measurements for skin texture quantification, at the preoperative stage, three, and six months postoperatively. A total of 8 patients were included. Repeated measurement was used to determine the lab data and VISIA measurement changes before and after the operation. Mauchly’s sphericity test was performed to determine whether the violation of sphericity occurs, and the Greenhouse–Geisser correction was used when the violation of sphericity occurs.

**Results:**

All the patients were female and generally healthy without systemic medical disease except the hyperthyroidism. The T4 and TSH levels were not significantly different before and after the thyroidectomy. In terms of the skin character measurements, the wrinkles, texture, pores, UV spots, and brown spots were not improved after thyroidectomy. A trend of improvement in spots, red area, and porphyrin was noted, although not statistically significant.

**Conclusions:**

Surgical removal of the thyroid gland in patients with hyperthyroidism does not improve the skin quality and texture in examinations via the VISIA system.

## Introduction

Hyperthyroidism is a medical condition of excess synthesis and secretion of the thyroid hormones by the thyroid gland. It is characterized by low serum thyroid-stimulating hormone (TSH) concentrations and raised serum concentrations of thyroid hormones: thyroxine (T_4_), tri-iodothyronine (T_3_), or both [1]. Signs and symptoms due to excess thyroid hormones include palpitations, fatigue, tremor, anxiety, disturbed sleep, weight loss, heat intolerance, sweating, and polydipsia [2–4]. In regard to the skin, a patient with hyperthyroidism may present with warm and moist skin, flushing of the face, erythema of the palms, hyperhidrosis of the palms and soles, and myxedema of the pretibial area. The epidermis is found to be relatively thin but not atrophic. In addition, scalp hair often has a downy texture and diffuse scalp hair thinning is often noted [5].

One of the treatment options for thyroid lesions with hyperthyroidism is surgery. Thyroidectomy surgeries are effective in treating hyperthyroidism patients with Grave’s disease, or those with toxic goiters and adenomas [6]. During clinical practice we have noticed that, some patients with hyperthyroidism, especially those with Grave’s disease, have an improvement in skin texture after thyroidectomy. Moreover, the improvement in skin texture can be observed within a few weeks after the operation. However, there is no evidence or study in the literature to proof this finding.

Dyspigmentation or dyschromia is a common complaint in the dermatology office. This is a broad term that includes disorders of melanin deposition, disorders of superficial vasculature, and disorders of both. The VISIA Complexion Analysis System (Canfield Imaging Systems, Fairfield, NJ) is a device used to measure and quantify a patient’s dyschromia, and it’s clinically applicable [7]. Via standard, ultraviolet, and cross polarized lighting, it generates a series of photographs to analyze skin characters, including spots, wrinkles, texture, pores, ultraviolet spots, brown spots, red area, and porphyrin. Spots, wrinkles, texture, and pores are analyzed through standard lighting. Ultraviolet spots and porphyrin are analyzed through ultraviolet lighting, and brown spots, red area through cross polarized lighting [7].

Therefore, our objective of this study is to evaluate and quantify the skin characters of patients with hyperthyroidism before and after thyroidectomy. The result of this study can help patients to better understand the skin characters or complaints throughout the course of surgical intervention for hyperthyroidism.

## Material and methods

**Fig. 1:**
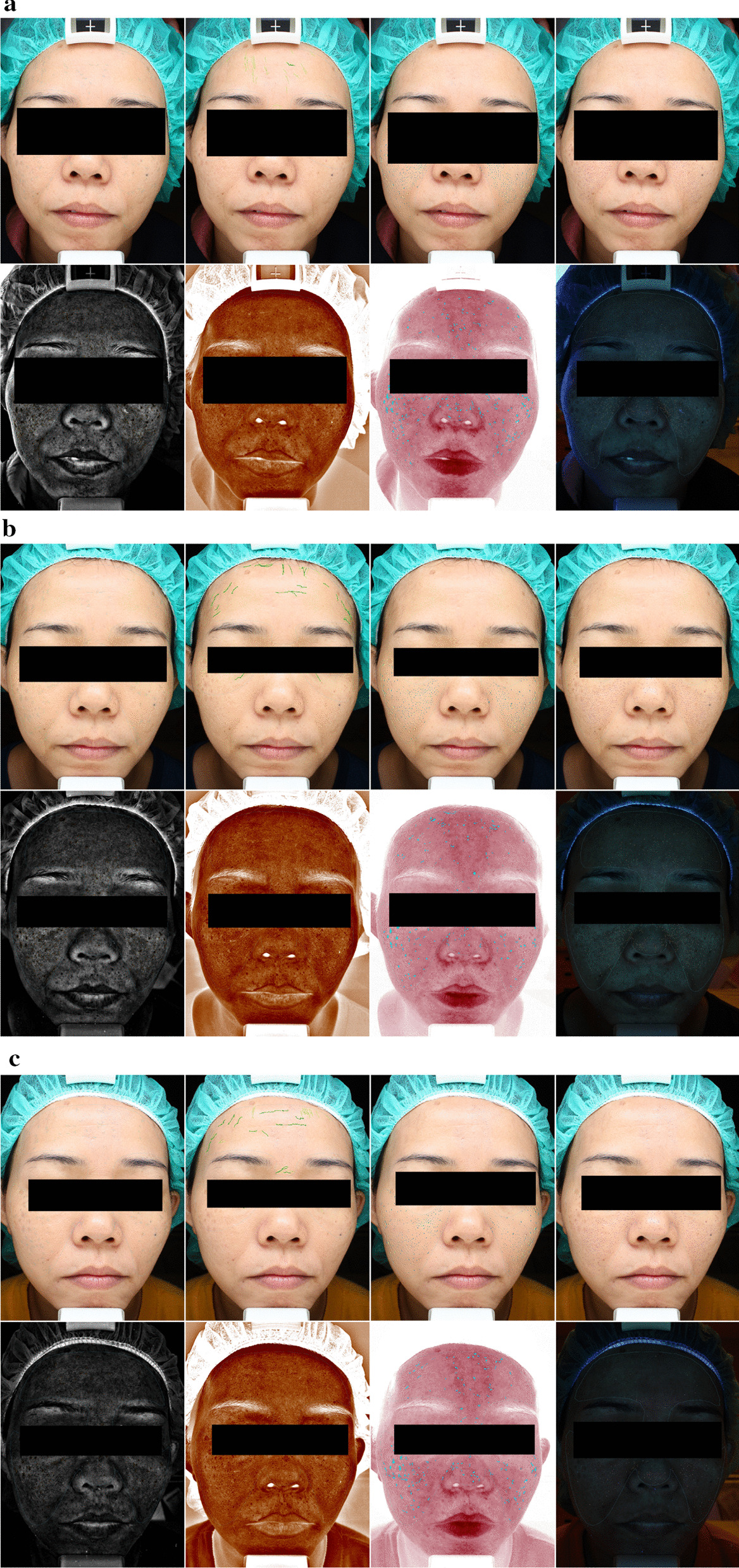
The VISIA photos of case number 1, taken at the preoperative stage (**a**), three months (**b**), and 6 months (**c**) postoperatively. The photos in the upper row, from left to right, represents signals of spots, wrinkles, texture, and pores. The photos in the lower row, from left to right, represents signals of UV spots, brown spots, red area, and porphyrin

This study was approved by the institutional review board (IRB) of the Kaohsiung Chang Gung Memorial Hospital, with the reference number of 201800309B0 and conformed to the ethical guidelines of the 1975 Declaration of Helsinki. Informed consent was obtained from all individual participants included in the study. We designed a prospective study to include patients with hyperthyroidism who received total thyroidectomy between March 1st, 2018 and February 28th, 2019. The patients would receive blood test for T4 and TSH analysis and VISIA measurements for skin texture quantification, at the preoperative stage (Fig. 1a), three months (Fig. 1b), and six months (Fig. 1c) postoperatively. Also, the skin texture improvement of each patient was evaluated by three independent plastic surgeons and a score of − 10 to 10 would be given. A total of 8 patients were included in this study, and one patient did not receive phlebotomy for T4 and TSH analysis. Therefore, we only have the lab work data of 7 patients in this study. Detailed patient information was retrieved from medical records, including information regarding the following variables: age; sex; co-morbidities, such as diabetes mellitus, hypertension, heart disease, renal disease, liver disease, and smoking status.

To evaluate the relationship between thyroidectomy and skin texture changes, the data of the patients were analyzed using the SPSS v.22 statistical software (IBM, Armonk, NY). Repeated measurement was used to determine the lab data and VISIA measurement changes before and after the operation. Sphericity is an important assumption of a repeated measures. It is the condition where the variances of the differences between all possible pairs of within-subject conditions are equal. If sphericity is violated, then the variance calculations may be distorted, which may lead to an invalid result. Mauchly’s sphericity test was performed to determine whether the violation of sphericity occurs. Should the sphericity be violated, the variance calculations could be distorted, then we would apply the Greenhouse–Geisser correction method to correct the distortion. A *p*-value of < 0.05 was considered statistically significant.

## Results

**Fig. 2:**
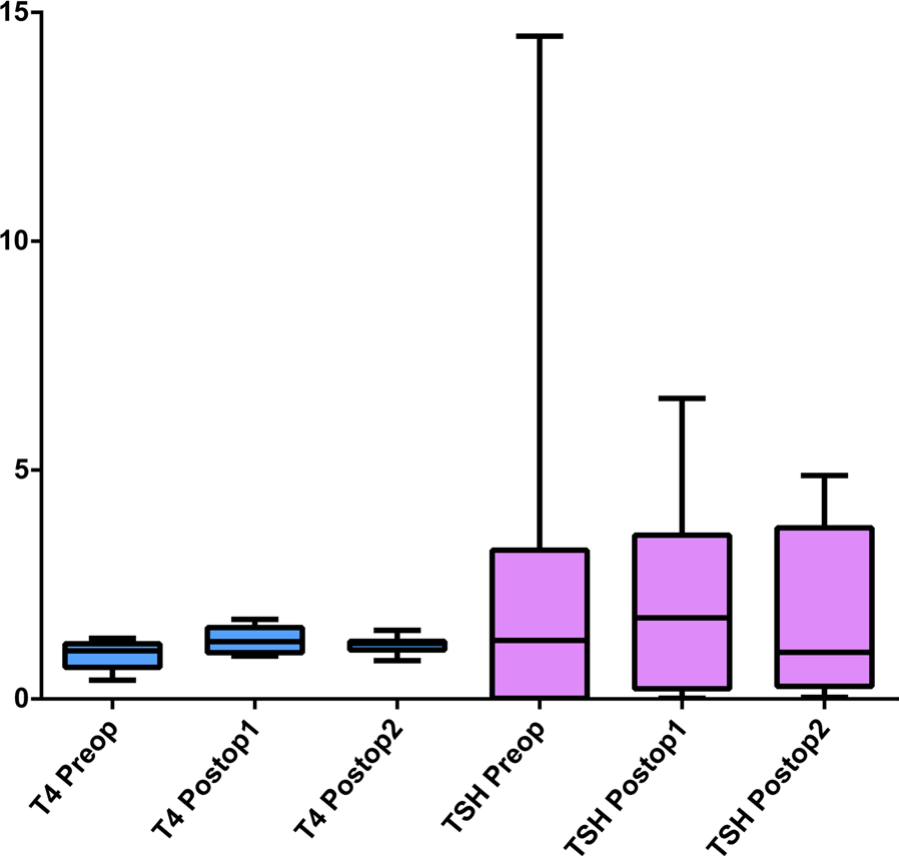
The T4 and TSH levels at the preoperative stage, three, and six months postoperatively. Postop1 = 3 months postoperatively; Postop2 = 6 months postoperatively

**Fig. 3:**
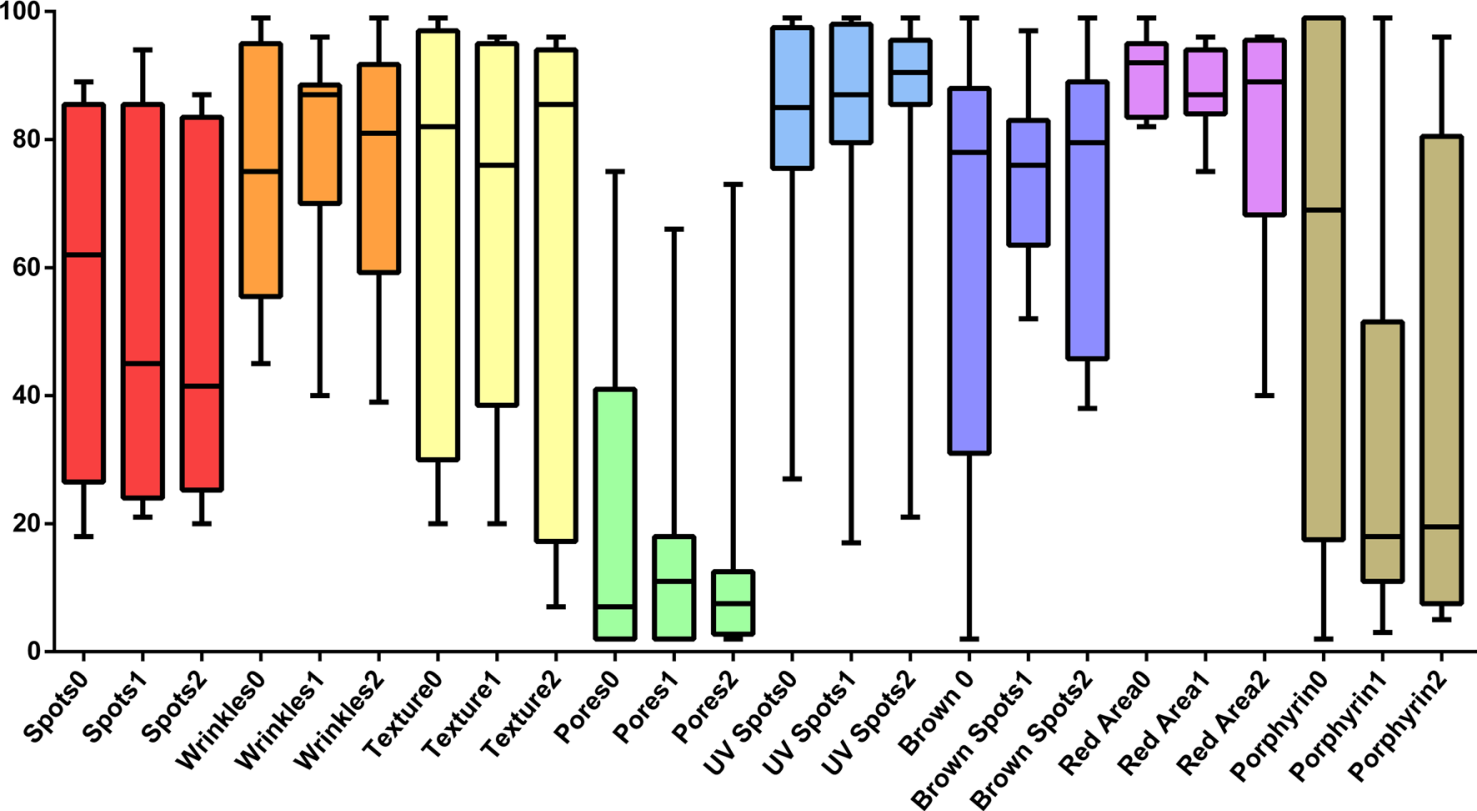
The VISIA measurements at the preoperative stage, three, and six months postoperatively. 0 = preoperative stage; 1 = 3 months postoperatively; 2 = 6 months postoperatively

**Fig. 4:**
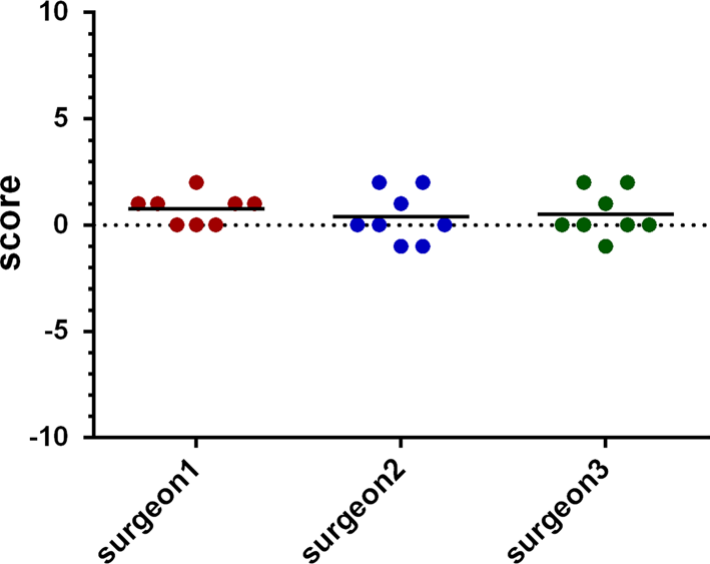
Scoring for skin texture improvement at 6 months postoperatively

The basic information for all patients that received total thyroidectomy for hyperthyroidism were summarized in Table 1. All the patients were female and generally healthy without systemic medical disease except the hyperthyroidism. One patient had medical history of atrial fibrillation, acute heart failure with pleural effusion, which was related to thyroid storm attack according to the chart record. The statistical analysis of lab data and measurements of the skin characters using the VISIA Complexion Analysis System before and after thyroidectomy were summarized in Table 2. One patient did not receive blood test for T4 and TSH analysis. The T4 (0.961 ± 0.332 vs. 1.304 ± 0.306 vs. 1.173 ± 0.202, *p* = 0.122) and TSH levels (2.899 ± 5.341 vs. 2.416 ± 2.398 vs. 1.289 ± 1.491, *p* = 0.613) were not significantly different before and after the thyroidectomy (Fig. 2). In terms of the skin character measurements, the wrinkles (73.75 ± 21.711 vs. 77.50 ± 18.174 vs. 76.00 ± 20.723, *p* = 0.857), texture (72.75 ± 32.897 vs. 70.63 ± 30.374 vs. 63.63 ± 39.049, *p* = 0.589), pores (23.50 ± 28.279 vs. 17.13 ± 20.829 vs. 15.13 ± 23.709, *p* = 0.783), UV spots (79.75 ± 23.499 vs. 80.50 ± 27.013 vs. 82.88 ± 25.430, *p* = 0.607), and brown spots (64.38 ± 38.112 vs. 75.88 ± 13.293 vs. 71.63 ± 23.003, *p* = 0.588) were not improved after thyroidectomy. However, we did notice a trend of improvement in spots (59.88 ± 28.180 vs. 53.38 ± 30.668 vs. 50.88 ± 28.412, *p* = 0.164), red area (90.50 ± 6.590 vs. 88.13 ± 6.896 vs. 81.13 ± 19.730, *p* = 0.140), and porphyrin (62.38 ± 37.713 vs. 34.50 ± 31.942 vs. 36.25 ± 37.909, *p* = 0.127), although not statistically significant (Fig. 3). The scoring for skin texture improvement at six months postop was summarized in Fig. 4. No significant improvement was reported by the independent reviewers.

**Table 1:** Demographic data of patients receiving thyroidectomy and VISIA system analysis

Patient No	Age	Sex	Smoker	Etiology of hyperthyroidism	Systemic disease (Except thyroid disease)
1	44	Female	No	Grave's disease	None
2	60	Female	No	Toxic thyroid nodules, papillary microcarcinoma	None
3	26	Female	No	Grave's disease	None
4	53	Female	No	Hashimoto thyroiditis	Atrial fibrillation; Acute heart failure with pleural effusion*
5	53	Female	No	Grave's disease	None
6	33	Female	No	Grave's disease	None
7	23	Female	No	Grave's disease	None
8	33	Female	No	Grave's disease	None

*May be hyperthyroidism related

**Table 2:** Statistical analysis of lab data and measurements of the skin characteristics using the VISIA Complexion Analysis System before and after thyroidectomy (n = 7 for T4 and TSH)

	Preoperative (Mean ± SD)	Postoperation 3 months (Mean ± SD)	Postoperation 6 months (Mean ± SD)	*p*-value
T4	0.961 ± 0.332	1.304 ± 0.306	1.173 ± 0.202	0.122
TSH	2.899 ± 5.341	2.416 ± 2.398	1.289 ± 1.491	0.613
Spots	59.88 ± 28.180	53.38 ± 30.668	50.88 ± 28.412	0.164
Wrinkles	73.75 ± 21.711	77.50 ± 18.174	76.00 ± 20.723	0.857
Texture	72.75 ± 32.897	70.63 ± 30.374	63.63 ± 39.049	0.589
Pores	23.50 ± 28.279	17.13 ± 20.829	15.13 ± 23.709	0.783
UV spots	79.75 ± 23.499	80.50 ± 27.013	82.88 ± 25.430	0.607
Brown spots	64.38 ± 38.112	75.88 ± 13.293	71.63 ± 23.003	0.588
Red area	90.50 ± 6.590	88.13 ± 6.896	81.13 ± 19.730	0.140
Porphyrin	62.38 ± 37.713	34.50 ± 31.942	36.25 ± 37.909	0.127

*SD* standard deviation, *T4* thyroxine, *TSH* thyroid-stimulating hormone, *UV* ultraviolet

## Discussion

The connections between thyroid diseases and it’s skin manifestations have been observed in early days [8]. Direct thyroid hormone actions on the skin can be observed on multiple aspects of cutaneous biology, including the dermis, epidermis, and hair. However, the thyroid hormone receptor superfamily, which mediates the direct action of thyroid hormones on the skin, is one of the members that is less understood regarding the skin [9]. In the case of hyperthyroidism or thyrotoxicosis, warm and moist skin that is hyperhydrosis, thin epidermis that is not atrophic, skin dyspigmentation, fine and soft hair, and Plummer’s nails can be observed. The warmth of the skin is resulted from increased cutaneous blood flow and the moisture reflects the underlying hypermetabolic status, which are both secondary to the hyperthyroidism [10, 11]. Clinical observation of the skin thinning in patients with hyperthyroidism is more complicated, because most hyperthyroidism results from Grave’s disease, in which autoimmune-mediated glycosaminoglycan can deposit in the dermis [12]. Generally, the interactions between the thyroid hormones and the thyroid hormone receptor superfamily of the skin are very complex.

However, thyroidectomy was not connected to improved skin texture postoperatively according to the VISIA measurements in our study. This may be due to the fact that there were no significant thyroid hormone level changes between the preoperative and postoperative periods for all the cases included in this study. Most thyroid diseases can be well controlled by existing medications. All the patients included in this study received antithyroid medication preoperatively and oral form thyroxine supplement postoperatively. Also, complaints or discomforts in terms of the skin were already heard and managed by our endocrinologist in the outpatient department setting. As a result, the thyroidectomy did not significantly influence the serum TSH and T4 level, and therefore did not significantly influence the skin metabolism. Another possible explanation may be the insufficient follow up periods, which was merely 6 months.

A specific strength of our study is the use of the VISIA system, which analyzes and quantifies the dyschromia of the patients’ skin preoperatively and postoperatively. However, this study has also several limitations. The most important limitation is the design and patient selection of the study. Although this study is prospective, we cannot find a group of hyperthyroidism patients without medical treatment. As a result, the effect of thyroidectomy on serum thyroid hormone level is masked by the antithyroid medication and the thyroxine supplement. The study is also limited by a small study population and not being able to distinct the different cause of hyperthyroidism. Also, the follow up may be insufficient. A more comprehensive, protocol-based prospective study with wider and more focused series of cases on a more specific category of patients is needed to address these issues.

## Conclusions

In conclusion, this study reveals that surgical removal of the thyroid gland in patients with hyperthyroidism does not improve the skin quality and texture in examinations via the VISIA system.

## Data Availability

De-idenfication data could be provided for academic purpose from the corresponding author on reasonable request.

## Abbreviations

TSH: Thyroid-stimulating hormone
T4: Thyroxine
T3: Tri-iodothyronine

## References

[1] De Leo S, Lee SY, Braverman LE (2016). Hyperthyroidism. Lancet (London, England).

[2] Goichot B, Caron P, Landron F, Bouee S (2016). Clinical presentation of hyperthyroidism in a large representative sample of outpatients in France: relationships with age, aetiology and hormonal parameters. Clin Endocrinol.

[3] Devereaux D, Tewelde SZ (2014). Hyperthyroidism and thyrotoxicosis. Emerg Med Clin North Am.

[4] Boelaert K, Torlinska B, Holder RL, Franklyn JA (2010). Older subjects with hyperthyroidism present with a paucity of symptoms and signs: a large cross-sectional study. J Clin Endocrinol metab.

[5] Lause M, Kamboj A, Fernandez Faith E (2017). Dermatologic manifestations of endocrine disorders. Transl Pediatr.

[6] van Soestbergen MJ, van der Vijver JC, Graafland AD (1992). Recurrence of hyperthyroidism in multinodular goiter after long-term drug therapy: a comparison with Graves' disease. J Endocrinol Invest.

[7] Goldsberry A, Hanke CW, Hanke KE (2014). VISIA system: a possible tool in the cosmetic practice. JDD.

[8] Ord WM (1878). On Myxoedema, a term proposed to be applied to an essential condition in the "Cretinoid" Affection occasionally observed in Middle-aged Women. Med Chir Trans.

[9] Safer JD (2012). Thyroid hormone action on skin. Curr Opin Endocrinol Diabetes Obes.

[10] Weiss M, Milman B, Rosen B, Zimlichman R (1993). Quantitation of thyroid hormone effect on skin perfusion by laser Doppler flowmetry. J Clin Endocrinol Metab.

[11] Pazos-Moura CC, Moura EG, Breitenbach MM, Bouskela E (1998). Nailfold capillaroscopy in hypothyroidism and hyperthyroidism: blood flow velocity during rest and postocclusive reactive hyperemia. Angiology.

[12] Holt PJ, Marks R (1977). The epidermal response to change in thyroid status. J Invest Dermatol.

